# Systemic glucocorticoids and the risk of breast cancer in a large nationwide case–control study

**DOI:** 10.1186/s13058-025-02071-0

**Published:** 2025-06-23

**Authors:** Manon Cairat, Morten Olesen, Elea Olivier, Anton Pottegård, Blánaid Hicks

**Affiliations:** 1 Paris-Saclay University, UVSQ, Inserm, Gustave Roussy, Exposome and Heredity Team, CESP, F-94805 Villejuif, France; 2 Clinical Pharmacology and Pharmacy, Department of Public Health, University of Southern Denmark, Odense, Denmark; 3 Centre for Public Health, Queen’s University Belfast, ICSB, Royal Victoria Hospital, Grosvenor Road, Belfast, BT12 6BA Northern Ireland

**Keywords:** Glucocortiocoids, Breast cancer, Registries

## Abstract

**Background:**

Concerns have been raised that long-term use of glucocorticoids may increase the risk of breast cancer, yet evidence is limited. Thus, this study investigated the association between systemic glucocorticoid use and breast cancer risk, overall and by breast cancer subtype and stage.

**Methods:**

A nationwide case–control study was conducted using the Danish healthcare registries. Women with invasive breast cancer between 2001 and 2018 (*n* = 67,829) were identified as cases. Each case was matched to 10 population controls on age and calendar time. Ever users of glucocorticoids were defined as women who filled at least 2 prescriptions and long-term users those who filled prescriptions equivalent to ≥ 1000 defined daily doses (DDDs). Conditional logistic regressions were performed to calculate odds ratios (ORs) and 95% confidence intervals for the associations between glucocorticoid use and breast cancer risk.

**Results:**

Twelve percent of women (*n* = 87,277) had ever been exposed to glucocorticoids and fewer than 1% were long-term users (*n* = 5,574). No association was found between ever use of glucocorticoids and breast cancer risk [OR = 1.00 (0.98—1.03)], compared with never use. However, an inverse association was observed between long-term glucocorticoid use and breast cancer risk [OR = 0.87 (0.77—0.97)], with suggestion of a slight dose–response relationship [OR per 500 DDDs = 0.96 (0.94—0.99)]. The associations were consistent across different tumour subtypes, estrogen receptor status, or clinical stage at diagnosis.

**Conclusion:**

The findings from this large nationwide study did not suggest a positive association between glucocorticoids and breast cancer risk.

**Supplementary Information:**

The online version contains supplementary material available at 10.1186/s13058-025-02071-0

## Introduction

Due to their divergent biological properties, synthetic glucocorticoids are commonly used to treat a variety of conditions, including allergies, rheumatologic disorders, respiratory, autoimmune and inflammatory diseases [1, 2]. While widely used, concerns have been raised that prolonged use of glucocorticoids may increase the risk of developing several cancers [3–5]. Indeed, glucocorticoids are known for their immunossupressives properties [6–8], which may allow tumour cells to escape from immune surveillance. Moreover, these drugs could promote insulin resistance and metabolic dysfunction [9–11], which have been associated with an increased risk of various cancers [12–14]. Despite this potential role in carcinogenesis, glucocorticoids may also have opposing effect on breast cancer carcinogenesis through inhibition of estrogens and inflammation [15]. Yet, limited studies on breast cancer have been conducted. Two studies from Northern Denmark reported no association with systemic glucocorticoids, however, they lacked results for specific breast cancer subtypes [16, 17].

This may be important because glucocorticoids could interact with estrogen receptors and glucocorticoid receptors in mammary epithelial cells [18]. In a subsequent study, performed by our group in the French E3N cohort, systemic glucocorticoids were associated with a lower risk of early-stage breast cancers, particularly for estrogen-receptor positive cancer, but elevations in risk for advanced-stage breast cancers [19]. Although experimental studies have documented the potential dual effects of glucocorticoids on breast cancer development [20–24], this was the first epidemiological study which examined the glucocorticoids-breast cancer associations, taking tumor stage or subtypes into account.

Thus, we used the nationwide Danish registries to investigate the association between systemic glucocorticoids use and breast cancer risk, overall and by breast cancer subtype and stage.

## Materials and methods

### Nationwide registry sources

A nested case–control study was conducted using data from the six following nationwide registries: the Danish Cancer Registry [25], the National Prescription Registry [26], the National Patient Registry [27], Registers in Statistics Denmark on educational level [28], the Danish Pathology Register [29] and the Civil Registration System [30, 31]. We described these registries in Appendix S1 (Additional file 1).

In Denmark, almost all medical care is funded by the Danish National Health Service, allowing comprehensive population-based register linkage studies that cover all residents of the country [32]. Data sources were linked by a unique personal identification number, assigned to all residents since 1968 [31]. All linkages were performed by Statistics Denmark, a government agency responsible for collecting and processing data for various statistical and scientific purposes**.**

### Selection of cases and controls

Breast cancer cases were retrieved from the Danish Cancer Registry. Codes for cancer diagnoses are described in Appendix S2 (additional file 1). All women with a histologically verified primary diagnosis of invasive breast cancer between January 1 st 2001 and December 31 st 2018 were defined as cases. The date of diagnosis corresponded to the index date. Only patients aged between ≥ 18 and < 85 years at the index date were included.We further excluded patients with any residency outside Denmark in the 10 years prior to the index date, which ensured that at least 10 years of follow-up for all study subjects. Since the prescription registry opened in 1995, it also ensuresa minimum of 5 years of prescription data. We also excluded women with any cancer diagnosis (except non-melanoma skin cancer) or mastectomy before the index date. The study sample was restriced to women with no prescription of systemic glucocorticoids between January 1, 1995th and December 31 st, 1995 in order to exclude those who likely began using these drugs before prescription data became available.

For each case, 10 controls among Danish women were matched by exact birth year and calendar time. The same selection criteria applied to both cases and controls. Controls were selected through risk set sampling and assigned the same index date as the matched case. Subjects were eligible to be selected as controls before they became cases, ensuring that the calculated ORs provide unbiased estimates of the incidence rate ratios that would be estimated from a cohort study using the underlying source population [33].

### Exposure

All prescriptions of systemic glucocorticoids were retrieved since January 1 st, 1996. Codes for drug exposure are listed in Appendix 2. “Ever users” were defined as women with at least two prescriptions between 1 st January 1996 and the index date. “Never users” (the reference category) were defined as those with 0—1 prescription, as a single prescription is unlikely to result in sufficient exposure to meaningfully affect breast cancer risk, which is typically associated with prolonged or repeated use. Exposure was also considered based on the cumulative number of defined daily doses (DDD). Women who filled prescriptions equivalent to ≥ 1000 DDDs of systemic glucocorticoids were considered as long-term users. In addition, the 5-most frequently prescribed glucocorticoids were individually analysed: betamethasone, methylprednisolone, prednisone, prednisolone, and hydrocortisone. Prescriptions filled in the year prior to the index date were exluded for all analyses to ensure a minimum latency period and to account for potential reverse causality [34].

### Covariates

Prescriptions of drugs suspected to modify breast cancer risk and likely to be associated with the use of systemic glucocorticoids were retrieved from the Prescription Registry. This include immunosuppressants, non-steroidal anti-inflammatory drugs and proton pump inhibitors. Women with at least two prescriptions of the drug of interest from 1995 to one year prior to the index date were defined as ever users. For oral contraceptives or hormone replacement therapy, recent users were defined as women with at least two prescriptions in the year immediately preceding the lagged index date. Former users were defined as women with at least two prescriptions between 1995 and the year preceding immediately the lagged index date but who were not recent users.

Diagnoses of comorbidities were retrieved from the Danish National Patient Registry. Comorbidities were defined as a primary or secondary discharge, outpatient diagnoses or by related medications. Alcohol-related diseases and chronic obstructive pulmonary disease were considered as proxies for heavy alcohol consumption and smoking, respectively. We also considered comorbidities requiring systemic glucocorticoid use including, asthma, rheumatoid arthritis, polymyalgia rheumatica/giant cell arthritis, psoriasis arthritis, ankylosing spondylitis, Crohn’s disease, ulcerative colitis, renal diseases, and multiple sclerosis. The Charlson comorbidity index score was categorized as follows: 0 (low), 1–2 (medium), or ≥ 3 (high), based on the prevalence of 19 chronic conditions [35, 36]. Information within one year prior to the index date was also disregarded for comorbidities. Information on educational level was obtained from the registries at Statistics Denmark and the Civil Registration System, using it as a crude measure of socioeconomic status (basic, medium, higher or unknown). Codes for covariates are listed in Appendix S2.

### Statistical analyses

The frequency and proportion of cases and controls were calculated within categories of exposure and covariates. Conditional logistic regression was used to estimate odds ratios (ORs) and 95% confidence intervals (95% CI) for the association between systemic glucocorticoid use and breast cancer risk.

Analyses were stratified by predefined categories of cumulative doses of systemic glucocorticoids (< 500, ≥ 500—< 1000, ≥ 1000—< 1500, ≥ 1500 DDDs) to explore potential dose–response associations. In all analyses, never use of systemic glucocorticoids (defined as having filled 0–1 prescriptions) served as the reference category. In analyses of individual glucocorticoids (i.e. betamethasone, methylprednisolone prednisone, triamcinolone, prednisolone and hydrocortisone), the reference class was never use of any systemic glucocorticoids. Models were adjusted for asthma, rheumatoid arthritis, polymyalgia rheumatica/giant cell arthritis, psoriasis arthritis, ankylosing spondylitis, Crohn’s disease, ulcerative colitis, renal diseases, multiple sclerosis, Charlson comorbidity index score, ever use of immunosuppressants, ever use of non-steroidal anti-inflammatory drugs, ever use of proton pump inhibitors, former and recent use of oral contraceptives and former and recent use of hormone replacement therapy.

We conducted various subgroup and sensitivity analyses. First, we explored the association between systemic glucocorticoids and breast cancer risk, stratified by histological type (ductal adenocarcinoma, lobular adenocarcinoma, and others), estrogen receptor (ER) status (ER-positive, ER-negative, and unknown), and clinical stage at diagnosis (localized, non-localized and unknown). Then, we performed stratified analyses based on age at the index date (< 55, ≥ 55—< 70 and ≥ 70). We also repeated the main analyses by varying minimum latency period, ranging from 0 to 2 years, to test the robustness of our results against different assumptions about the minimum latency period [34]. Lastly, we restricted our analysis to women diagnosed with inflammatory bowel diseases (Crohn’s disease or ulcerative colitis) and those diagnosed with rheumatoid arthritis to address indication bias. All statistical analyses were conducted using STATA version 18.

**Fig. 1:**
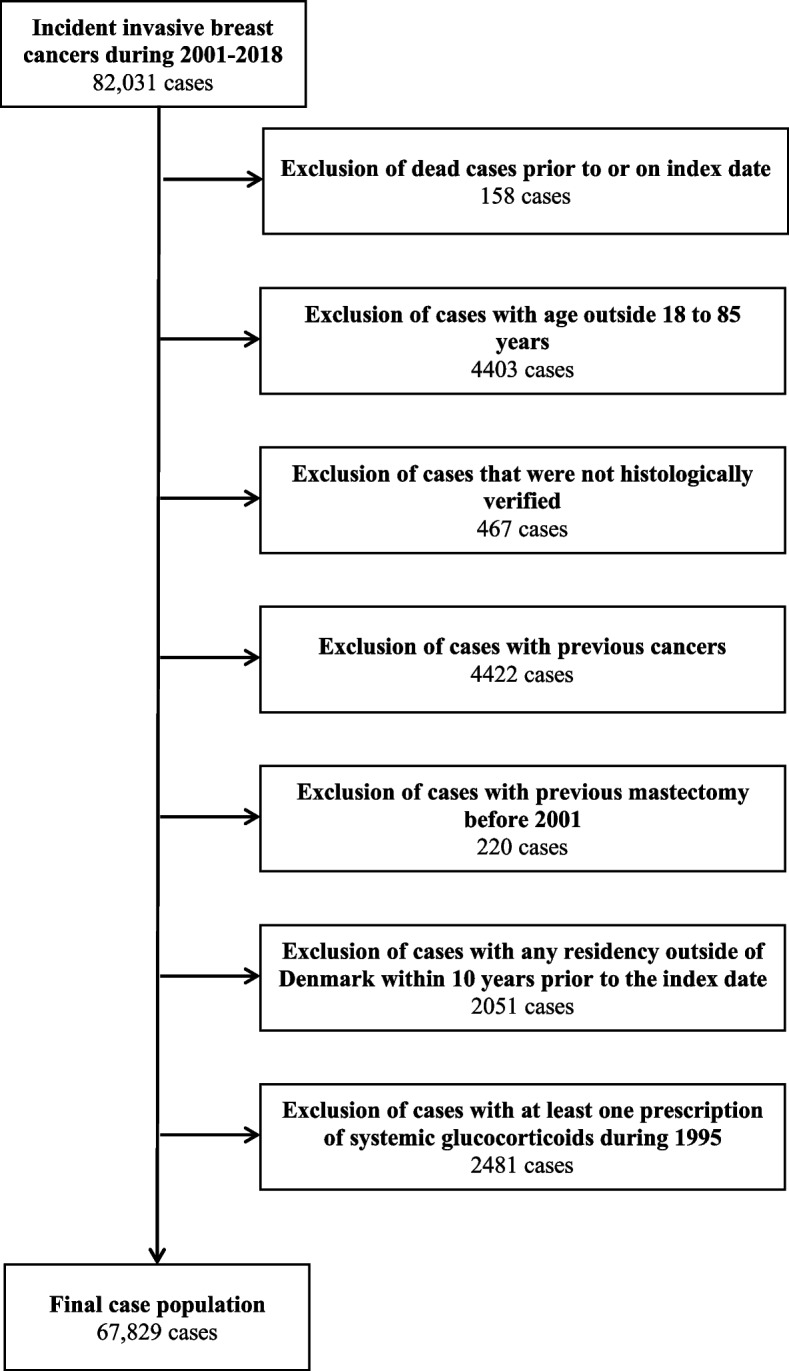
Flow-chart of the selection of cases

## Results

The study population included 67,829 breast cancer cases and 678,290 controls (Fig. 1). The majority of the cases were ductal adenocarcinomas (75%), followed by lobular adenocarcinomas (13%) and other breast cancers (12%). Among cases, 49,131 had data on ER status, with 79% being ER-positive and 19% ER-negative. Of the 54,661 cases with stage information, 56% were localized, while 43% were non-localized. The characteristics of the study population are presented in Table 1. The median age at index date was 62 years (interquartile range, 53—70). Differences in characteristics at index date between cases and controls were generally minor, with the exception of a higher use of hormone replacement therapy among cases compared to controls. At the index date, 12% of women (for both cases and controls) had filled at least one prescription for systemic glucocorticoids, while less than 1% were long-term users of systemic glucocorticoids.

**Table 1:** Characteristics of breast cancer cases and matched controls

	**Cases, n = 67,829**	**Controls, n = 678,290**
**Age, median (IQR, years)**	62 (53–70)	62 (53–70)
**Breast cancer histological type**		
Ductal adenocarcinoma	50,849 (75%)	NA
Lobular adenocarcinoma	8,671 (13%)	NA
Other histologies	8,309 (12%)	NA
**Breast cancer ER status**		
ER-	9,261 (14%)	NA
ER +	39,870 (59%)	NA
Unknown	18,698 (28%)	NA
**Breast cancer stage**		
Localized	30,898 (46%)	NA
Non localized	23,763 (35%)	NA
Unknown	13,168 (19%)	NA
**Use of systemic glucocorticoids**		
Never	59,868 (88%)	598,974 (88%)
Ever^a^	7,961 (12%)	79,316 (12%)
Long-term^b^	439 (0.65%)	5,135 (0.76%)
Cumulative DDDs, median (IQR)	87 (43–250)	90 (45–272)
**Ever use of other drugs**^a^		
Immunosuppressants	1,023 (1.5%)	11,662 (1.7%)
Nonsteroidal anti-inflammatory drugs	40,210 (59%)	395,844 (58%)
Proton pump inhibitors	4,011 (5.9%)	40,581 (6.0%)
Raloxifene	138 (0.2%)	2,150 (0.3%)
Recent oral contraceptives	2,718 (4.0%)	21,520 (3.2%)
Former oral contraceptives	13,451 (20%)	127,595 (19%)
Recent hormone replacement therapy	7,564 (11%)	42,210 (6.2%)
Former hormone replacement therapy	17,649 (26%)	141,697 (21%)
**Comorbidities**		
Alcohol related diseases	2,187 (3.2%)	19,770 (2.9%)
Chronic obstructive pulmonary disease	14,209 (21%)	136,968 (20%)
Asthma	1,758 (2.6%)	16,764 (2.5%)
Rheumatoid arthritis	742 (1.1%)	8,771 (1.3%)
Polymyalgia rheumatica/Giant cell arthritis	375 (0.6%)	4,111 (0.6%)
Psoriasis arthritis	171 (0.3%)	1,762 (0.3%)
Ankylosing spondylitis	51 (0.1%)	505 (0.1%)
Crohn’s disease	207 (0.3%)	2,112 (0.3%)
Ulcerative colitis	452 (0.7%)	4,764 (0.7%)
Renal diseases	732 (1.1%)	7,149 (1.1%)
Multiple sclerosis	305 (0.4%)	2,662 (0.4%)
Adrenal insufficiency	37 (0.1%)	440 (0.1%)
**Charlson Comorbidity Index**		
None (Score = 0)	53,622 (79%)	539,151 (79%)
Low (Score = 1)	8,551 (13%)	86,992 (13%)
Medium (Score = 2)	3,319 (4.9%)	30,450 (4.5%)
High (Score ≥ 3)	2,337 (3.4%)	21,697 (3.2%)
**Highest achieved education**		
Short (7–10 years)	22,805 (34%)	247,786 (37%)
Medium (11–12 years)	25,010 (37%)	243,200 (36%)
Long (≥ 13 years)	18,503 (27%)	171,669 (25%)
Missing or unknown	1,511 (2.2%)	15,635 (2.3%)

*DDD* Defined Daily dose, *ER* Estrogen Receptor, *IQR* InterQuartile Range

^a^ Ever use was considered when filled ≥ 2 prescriptions more than 1 years prior to the index date

^b^ Long-term use was considered when filled prescriptions was equivalent to ≥ 1,000 DDDs

The associations between systemic glucocorticoid use and breast cancer risk are shown in Table 2. In fully-adjusted models, ever use of systemic glucocorticoids was not associated with breast cancer risk [OR = 1.00 (0.98—1.03)], while long-term use (≥ 1000 DDDs) was associated with a slightly decreased breast cancer risk [OR = 0.87 (0.77—0.97)], compared to never use. A slight dose–response relationship was found [OR_per 500 DDDs_ = 0.96 (0.94—0.99)]. Estimates were generally similar across long-term use of individual glucocorticoids [OR_methylprednisolone_ = 0.72 (0.37—1.38), OR_prednisone_ = 0.84 (0.60—1.17), OR_prednisolone_ = 0.86 (0.75—0.98), OR_hydrocortisone_ = 1.03 (0.47—2.28)].

**Table 2:** Associations between systemic glucocorticoid use and breast cancer risk, overall and by type of glucocorticoids

	**n case**	**n controls**	**OR (95% CI)**^**a**^	**OR (95% CI)**^b^
**All systemic glucocorticoids**				
**Use categories**				
Never use	59,868	598,974	1.00 (ref.)	1.00 (ref.)
Ever use	7,961	79,316	1.00 (0.98–1.03)	1.00 (0.98–1.03)
Long-term use	439	5,135	0.86 (0.78–0.94)	0.87 (0.77–0.97)
**Cumulative DDDs**				
Never use	59,868	598,974	1.00 (ref.)	1.00 (ref.)
< 500	6,905	67,431	1.02 (1.00–1.05)	1.02 (0.99–1.04)
≥ 500—< 1000	617	6,750	0.91 (0.84–0.99)	0.92 (0.85–1.01)
≥ 1000—< 1500	209	2,441	0.86 (0.74–0.99)	0.87 (0.75–1.01)
≥ 1500	230	2,694	0.85 (0.75–0.98)	0.88 (0.76–1.01)
* OR per 500 DDDs*	7,961	79,316	0.96 (0.94–0.98)	0.96 (0.94–0.99)
**Type of glucocorticoids**^c^				
**Betamethasone**				
Ever use	2,403	23,016	1.04 (1.00–1.09)	1.04 (0.99–1.08)
Long-term use	(n < 5)	44	(-)	(-)
**Methylprednisolone**				
Ever use	1,107	10,939	1.01 (0.95–1.08)	1.00 (0.93–1.06)
Long-term use	10	125	0.80 (0.42–1.53)	0.72 (0.37–1.38)
**Prednisone**				
Ever use	539	5,935	0.92 (0.84–1.00)	0.90 (0.82–0.99)
Long-term use	42	511	0.83 (0.61–1.15)	0.84 (0.60–1.17)
**Prednisolone**				
Ever use	3,730	38,098	0.98 (0.95–1.02)	0.99 (0.95–1.03)
Long-term use	339	3,980	0.85 (0.76–0.95)	0.86 (0.75–0.98)
**Hydrocortisone**				
Ever use	18	239	0.76 (0.47–1.24)	0.83 (0.47–1.45)
Long-term use	9	102	0.93 (0.47–1.85)	1.03 (0.47–2.28)

*Abbreviations*: *CI* confidence interval, *DDD* defined daily dose, *OR* Odds ratio dose

^a^ Adjusted for age and calendar time (by risk-set matching and the conditional analysis)

^b^ Adjusted for age and calendar time (by risk-set matching and the conditional analysis), asthma, rheumatoid arthritis, polymyalgia rheumatica/giant cell arthritis, psoriasis arthritis, ankylosing spondylitis, Crohn’s disease, ulcerative colitis, renal diseases, multiple sclerosis, Charlson comorbidity index score, ever use of immunosuppressants, ever use of non-steroidal anti-inflammatory drugs, ever use of proton pump inhibitors, former and recent use of oral contraceptives and former and recent use of hormone replacement therapy

^c^The reference class was never use of any systemic glucocorticoids

The association between systemic glucocorticoids and breast cancer risk were consistent across ER status (P_homogeneity_ ≥ 0.22, Table 3), tumor stage (P_homogeneity_ ≥ 0.22, Table 4) and age at index date (P_homogeneity_ ≥ 0.11, Supplementary Table S1). However, the inverse association were more pronounced for ductal adenocarcinoma [OR_long-term_ use = 0.80 (0.70—0.92)] and lobular adenocarcinoma [OR_long-term_ use = 0.81 (0.59—1.13)], compared to other histological types [OR_long-term_ use = 1.25 (0.96—1.63)] (P_homogeneity_ = 0.04, Supplementary Table S2). Changing the minimum latency period to 0 or 2 years instead of 1 year (main analysis) did not affect the estimates (Supplementary Table S3). Finally, estimates remained consistent after restricting the study sample to women diagnosed with inflammatory bowel diseases or rheumatoid arthritis (Supplementary Table S4).

**Table 3:** Associations of systemic glucocorticoid use with risk of breast cancer by estrogen receptor status

	**ER + **			**ER-**			**Unknown**			
	**n case**	**n controls**	**OR (95% CI)**^**a**^	**n case**	**n controls**	**OR (95% CI)**^**1**^	**n case**	**n controls**	**OR (95% CI)**^**a**^	***P***_***heterogeneity***_
**Use categories**										
Never use	35,249	352,811	1.00 (ref.)	8,216	82,413	1.00 (ref.)	16,403	163,750	1.00 (ref.)	
Ever use	4,621	45,889	1.02 (0.99–1.06)	1,045	10,197	1.01 (0.94–1.09)	2,295	23,230	0.97 (0.92–1.02)	0.24
Long-term use	261	2,987	0.92 (0.79–1.07)	61	633	0.94 (0.68–1.28)	117	1,515	0.74 (0.59–0.93)	0.22
**Cumulative DDDs**										
Never use	35,249	352,811	1.00 (ref.)	8,216	82,413	1.00 (ref.)	16,403	163,750	1.00 (ref.)	
< 500	4,008	38,924	1.03 (1.00–1.07)	910	8,674	1.03 (0.96–1.11)	1,987	19,833	0.98 (0.93–1.03)	
≥ 500—< 1000	352	3,978	0.92 (0.82–1.03)	74	890	0.79 (0.61–1.01)	191	1,882	1.00 (0.85–1.17)	
≥ 1000—< 1500	131	1,434	0.96 (0.79–1.15)	24	305	0.73 (0.47–1.13)	54	702	0.76 (0.57–1.02)	
≥ 1500	130	1,553	0.90 (0.75–1.09)	37	328	1.06 (0.74–1.52)	63	813	0.76 (0.58–1.00)	
* OR per 500 DDDs*	4,621	45,889	0.97 (0.94–1.01)	1,045	10,197	0.98 (0.91–1.06)	2,295	23,230	0.94 (0.89–0.99)	0.56

*Abbreviations*: *CI* confidence interval, *ER* estrogen receptor, *DDD* defined daily dose, *OR* Odds ratio

^a^Adjusted for age, calendar time (by risk-set matching and the conditional analysis), asthma, rheumatoid arthritis, polymyalgia rheumatica/giant cell arthritis, psoriasis arthritis, ankylosing spondylitis, Crohn’s disease, ulcerative colitis, renal diseases, multiple sclerosis, Charlson comorbidity index score, ever use of immunosuppressants, ever use of non-steroidal anti-inflammatory drugs, ever use of proton pump inhibitors, former and recent use of oral contraceptives and former and recent use of hormone replacement therapy

**Table 4:** Associations of systemic glucocorticoid use with risk of breast cancer by tumor stage

	**Localized**			**Non localized**			**Unknown**			
	**n case**	**n controls**	**OR (95% CI)**^**1**^	**n case**	**n controls**	**OR (95% CI)**^**1**^	**n case**	**n controls**	**OR (95% CI)**^**1**^	***P***_***heterogeneity***_
**Use categories**										
Never use	26,814	269,193	1.00 (ref.)	20,948	209,105	1.00 (ref.)	12,106	120,676	1.00 (ref.)	
Ever use	4,084	39,787	1.02 (0.98–1.06)	2,815	28,525	1.01 (0.96–1.05)	1,062	11,004	0.96 (0.89–1.03)	0.31
Long-term use	207	2,613	0.79 (0.67–0.93)	165	1,863	0.89 (0.74–1.08)	67	659	1.10 (0.81–1.49)	0.22
**Cumulative DDDs**										
Never use	26,814	269,193	1.00 (ref.)	20,948	209,105	1.00 (ref.)	12,106	120,676	1.00 (ref.)	
< 500	3,570	33,885	1.03 (0.99–1.07)	2,437	24,260	1.01 (0.97–1.06)	898	9,286	0.96 (0.89–1.03)	
≥ 500—< 1000	307	3,289	0.92 (0.81–1.04)	213	2,402	0.93 (0.80–1.08)	97	1,059	0.91 (0.73–1.14)	
≥ 1000—< 1500	96	1,194	0.80 (0.65–0.99)	80	879	0.97 (0.76–1.23)	33	368	0.90 (0.62–1.30)	
≥ 1500	111	1,419	0.79 (0.64–0.96)	85	984	0.92 (0.73–1.16)	34	291	1.20 (0.82–1.75)	
* OR per 500 DDDs*	4,084	39,787	0.95 (0.91–0.99)	2,815	28,525	0.98 (0.93–1.02)	1,062	11,004	0.99 (0.92–1.08)	0.45

*Abbreviations*: *OR* Odds ratio, *CI* confidence interval, *DDD* defined daily dose

^1^ Adjusted for age and calendar time (by risk-set matching and the conditional analysis)

^2^ Adjusted for age, calendar time (by risk-set matching and the conditional analysis), asthma, rheumatoid arthritis, polymyalgia rheumatica/giant cell arthritis, psoriasis arthritis, ankylosing spondylitis, Crohn’s disease, ulcerative colitis, renal diseases, multiple sclerosis, Charlson comorbidity index score, ever use of immunosuppressants, ever use of non-steroidal anti-inflammatory drugs, ever use of proton pump inhibitors, former and recent use of oral contraceptives and former and recent use of hormone replacement therapy

## Discussion

In this large nationwide registry-based study, we observed a small inverse association between long-term use of systemic glucocorticoids and breast cancer risk, also exhibiting a dose response relationship.

This is the largest study to evaluate the glucocorticoids-breast cancer associations to date. The principal strength of the present study is the use of nationwide registries known for their high validity [37, 38], with complete coverage of an entire nation, that allowed us to capture histologically verified breast cancer cases and risk-set sampling of controls with low risk of selection bias. Furthermore, the prospective design, combined with data from a drug prescription database, allowed us to identify systemic glucocorticoid exposure over a period of up to 23 years.

This approach allowed us to minimize any potential differential recall bias between cases and controls, while ensuring accurate and precise information on exposure. We were also able to adjust our models for socioeconomic parameters, use of other drugs and comorbidities and to minimize potential confounding by indication when restricting our analyses to women with a diagnosis of inflammatory bowel diseases and rheumatoid arthritis. It is important to acknowledge several limitations. Firstly, we did not have data on compliance and adherence to dispensed systemic glucocorticoids. However it is likely that this may be less of a concern for long-term users of glucocorticoids. Second, we used chronic obstructive pulmonary disease and alcohol-related diseases as proxies for heavy smoking and alcohol consumption [39, 40], both of which are associated with glucocorticoid use. However residual confounding may still be present due to lack of information on these specific factors. We were unable to adjust for important risk factors of breast cancer such as obesity and physical activity. These factors might also be associated with glucocorticoid use, either positively or inversely, and uncontrolled confounding from these factors could bias our results. Finally, we could not account for surveillance bias which might explain the inverse association observed. The findings reported in this paper are consistent with a previous study, performed by our group, suggesting a lower breast cancer risk with long-term exposure, with a trend for a dose–response relationship [19]. In addition, a trend for an inverse association was also found in a Norwegian drug-wide association study [41] and three other prospective studies, although they were not specifically designed to evaluate glucocorticoids-breast cancer associations [42–44]. Previous population-based case–control Danish studies reported a null association between systemic glucocorticoid use and invasive breast cancer risk, and no dose–response relationship [16, 17]. Of note, these previous studies had relative short follow-up and lacked power to properly evaluate glucocorticoids-breast cancer associations according to cumulative exposure.

Systemic glucocorticoids have been suggested to specifically lower the risk of ER + tumours. Indeed, these drugs have been shown shown to have preventive effects on breast cancer by stimulating the expression of sulfotransferase SULT1E1, which plays a role in deactivating estrogens [45]. Additionally, experimental models found that the expression of the glucocorticoid receptor was associated with improved breast cancer prognosis, particularly for ER + tumours, and that activation of the glucocorticoid receptor may reduce estrogen-induced cell proliferation in ER + breast cancer [18]. However, our current study suggests that the associations between glucocorticoids and breast cancer risk did not differ by ER status. Other potential mechanisms leading to a decreased breast cancer risk, regardless of the ER status, could include the effects of glucocorticoids on angiogenesis [21], or the inhibition of inflammatory and growth factors [22, 23]. Of note, the inverse association observed with long-term glucocorticoid use might be influenced by selection bias, as individuals with long-term exposure may represent a selected subgroup with a lower baseline risk. Additionally, chronic conditions, common in long-term users, may reduce cancer screening or detection, potentially leading to a spurious protective effect. These biases limit our ability to draw definitive conclusions[46, 47].

The only previous study that examined the glucocorticoid-breast cancer association by tumour stage found that systemic glucocorticoids were inversely associated with the risk of stage 1 or stage 2 tumours, but positively associated with the risk of stage 3 and 4 breast cancers [19]. Our current study found no such difference by tumor stage, although the inverse association observed between glucocorticoids and breast cancer risk was slightly stronger for localized breast cancer, compared to non-localized breast cancer. To the best of our knowledge, the is no known biological mechanism that may explain this potential heterogeneity by tumour stage.

## Conclusion

The findings from this large nationwide nested case–control did not suggest a positive association between glucocorticoids and breast cancer risk. Findings were consistent across ER status and tumour stage.

## Supplementary Information

Supplementary Material 1.

## Data Availability

This study is based on anonymized registry data located on a secure platform at Statistics Denmark, which can be accessed given the relevant data permits. Further information is available from the corresponding author upon request.

## Abbreviations

CI: Confidence interval
DDD: Defined daily dose
ER: Estrogen Receptor
OR: Odd ratio
