# Supplementary material for: Systemic glucocorticoids and the risk of breast cancer in a large nationwide case–control study
Source: Breast Cancer Res. 2025 Jun 23;27:114. doi: 10.1186/s13058-025-02071-0 (PMC12183910; doi:10.1186/s13058-025-02071-0)
Supplement: Supplementary file 1 — Supplementary Material 1. [file 13058_2025_2071_MOESM1_ESM.pdf]

Supplementary Material

Systemic glucocorticoids and the risk of breast cancer in a large nationwide case-control study

Cairat et al

Table of Contents

Table S1	Table S1. Associations of systemic glucocorticoid use with risk of breast cancer, stratified by age at index date.	Pg 2
Table S2	Associations of systemic glucocorticoid use with risk of breast cancer by histological type	Pg 3
Table S3	Association between systemic glucocorticoid use and invasive breast cancer risk with exposure with no lag period, 1-year lag and 2-year lag.	Pg 4
Table S4	Associations between systemic glucocorticoid use and breast cancer risk restricted to women with a diagnosis of rheumatoid arthritis and to women with a diagnosis of inflammatory bowel diseases.	Pg 5
Appendix 1	Danish Nationwide Health Registries	Pg 6
Appendix 2	Codes and definitions	Pg 7

Table S1. Associations of systemic glucocorticoid use with risk of breast cancer, stratified by age at index date.

	Age <55 years			Age ≥55 and <70 years			Age ≥70 years			
	n case	n controls	OR (95% CI)[1]	n case	n controls	OR (95% CI)[1]	n case	n controls	OR (95% CI)[1]	$P_{heterogeneity}$
**Use categories**										
Never use	18,409	183,125	1.00 (ref.)	26,365	264,868	1.00 (ref.)	15,094	150,981	1.00 (ref.)	
Ever use	1,399	14,955	0.95 (0.89-1.01)	3,581	34,592	1.03 (0.99-1.07)	2,981	29,769	1.00 (0.95-1.04)	0.10
Long-term use	37	543	0.69 (0.49-0.99)	143	1,743	0.79 (0.65-0.95)	259	2,849	0.97 (0.82-1.14)	0.11
**Cumulative DDDs**										
Never use	18,409	183,125	1.00 (ref.)	26,365	264,868	1.00 (ref.)	15,094	150,981	1.00 (ref.)	
<500	1,283	13,603	0.95 (0.90-1.01)	3,225	30,491	1.04 (1.00-1.09)	2,397	23,337	1.01 (0.96-1.06)	
≥500 - <1000	79	809	1.01 (0.79-1.28)	213	2,358	0.88 (0.76-1.01)	325	3,583	0.93 (0.83-1.06)	
≥1000 - <1500	21	269	0.81 (0.52-1.28)	71	799	0.86 (0.67-1.11)	117	1,373	0.89 (0.73-1.09)	
≥1500	16	274	0.62 (0.37-1.04)	72	944	0.75 (0.59-0.96)	142	1,476	1.03 (0.86-1.24)	
OR per 500 DDDs	1,399	14,955	0.92 (0.84-1.00)	3,581	34,592	0.94 (0.90-0.98)	2,981	29,769	0.99 (0.95-1.03)	0.11

Abbreviations: CI, confidence interval; DDD, defined daily dose; OR, Odds ratio.

[1] Adjusted for age, calendar time (by risk-set matching and the conditional analysis), asthma, rheumatoid arthritis, polymyalgia rheumatica/ giant cell arthritis, psoriasis arthritis, ankylosing spondylitis, Crohn's disease, ulcerative colitis, renal diseases, multiple sclerosis, Charlson comorbidity index score, ever use of immunosuppressants, ever use of non-steroidal anti-inflammatory drugs, ever use of proton pump inhibitors, former and recent use of oral contraceptives and former and recent use of hormone replacement therapy .

Table S2. Associations of systemic glucocorticoid use with risk of breast cancer by histological type.

	Ductal adenocarcinoma			Lobular adenocarcinoma			Other histologies			
	n case	n controls	OR (95% CI)[1]	n case	n controls	OR (95% CI)[1]	n case	n controls	OR (95% CI)[1]	$P_{heterogeneity}$
**Use categories**										
Never use	45,044	450,533	1.00 (ref.)	7,596	75,938	1.00 (ref.)	7,228	72,503	1.00 (ref.)	
Ever use	5,805	57,957	1.00 (0.97-1.03)	1,075	10,772	1.01 (0.94-1.09)	1,081	10,587	1.03 (0.96-1.11)	0.72
Long-term use	297	3,593	0.80 (0.70-0.92)	54	749	0.81 (0.59-1.13)	88	793	1.25 (0.96-1.63)	0.04
**Cumulative DDDs**										
Never use	45,044	450,533	1.00 (ref.)	7,596	75,938	1.00 (ref.)	7,228	72,503	1.00 (ref.)	
<500	5,078	49,570	1.01 (0.98-1.05)	945	9,108	1.03 (0.96-1.11)	882	8,753	1.02 (0.94-1.10)	
≥500 - <1000	430	4,794	0.89 (0.80-0.99)	76	915	0.87 (0.68-1.11)	111	1,041	1.12 (0.91-1.39)	
≥1000 - <1500	149	1,692	0.88 (0.74-1.05)	25	353	0.76 (0.50-1.16)	35	396	0.94 (0.65-1.35)	
≥1500	148	1,901	0.79 (0.66-0.94)	29	396	0.82 (0.55-1.22)	53	397	1.40 (1.03-1.91)	
OR per 500 DDDs	5,805	57,957	0.94 (0.91-0.97)	1,075	10,772	0.94 (0.87-1.02)	1,081	10,587	1.07 (1.01-1.14)	<0.01

Abbreviations: CI, confidence interval; DDD, defined daily dose; OR, Odds ratio.

[1] Adjusted for age, calendar time (by risk-set matching and the conditional analysis), asthma, rheumatoid arthritis, polymyalgia rheumatica/ giant cell arthritis, psoriasis arthritis, ankylosing spondylitis, Crohn’s disease, ulcerative colitis, renal diseases, multiple sclerosis, Charlson comorbidity index score, ever use of immunosuppressants, ever use of non-steroidal anti-inflammatory drugs, ever use of proton pump inhibitors, former and recent use of oral contraceptives and former and recent use of hormone replacement therapy..

Table S3. Association between systemic glucocorticoid use and invasive breast cancer risk with exposure with no lag period, 1-year lag and 2-year lag.

	Lag 0 year			Lag 1 year			Lag 2 years		
	n case	n controls	OR (95% CI)[1]	n case	n controls	OR (95% CI)[1]	n case	n controls	OR (95% CI)[1]
**All systemic glucocorticoids**									
Ever use	8,661	86,247	1.00 (0.98-1.03)	7,961	79,316	1.00 (0.98-1.03)	7,250	72,433	1.00 (0.97-1.03)
Long-term use	517	6,037	0.87 (0.78-0.97)	439	5,135	0.87 (0.77-0.97)	377	4,355	0.88 (0.78-1.00)
**Cumulative DDDs**									
Never use	59,168	592,043	1.00 (ref.)	59,868	598,974	1.00 (ref.)	60,579	605,857	1.00 (ref.)
<500	7,443	72,630	1.02 (0.99-1.04)	6,905	67,431	1.02 (0.99-1.04)	6,338	62,102	1.01 (0.98-1.04)
500-1000	701	7,580	0.93 (0.86-1.01)	617	6,750	0.92 (0.85-1.01)	535	5,976	0.91 (0.83-1.00)
1000-1500	247	2,836	0.88 (0.77-1.01)	209	2,441	0.87 (0.75-1.01)	185	2,132	0.89 (0.76-1.04)
>1500	270	3,201	0.86 (0.76-0.98)	230	2,694	0.88 (0.76-1.01)	192	2,223	0.89 (0.77-1.04)
OR per 500 DDDs	8,661	86,247	0.96 (0.94-0.99)	7,961	79,316	0.96 (0.94-0.99)	7,250	72,433	0.97 (0.94-1.00)

Abbreviations: OR, Odds ratio; CI, confidence interval; DDD, defined daily dose
[1] Adjusted for age, calendar time (by risk-set matching and the conditional analysis), asthma, rheumatoid arthritis, polymyalgia rheumatica/ giant cell arthritis, psoriasis arthritis, ankylosing spondylitis, Crohn’s disease, ulcerative colitis, renal diseases, multiple sclerosis, Charlson comorbidity index score, ever use of immunosuppressants, ever use of non-steroidal anti-inflammatory drugs, ever use of proton pump inhibitors, former and recent use of oral contraceptives and former and recent use of hormone replacement therapy..

Table S4. Associations between systemic glucocorticoid use and breast cancer risk restricted to women with a diagnosis of rheumatoid arthritis and to women with a diagnosis of inflammatory bowel diseases

	n case/control	n case/control	OR (95% CI)[1]
**Among women with rheumatoid arthritis**			
**Use categories**			
Never use	486	5,096	1.00 (ref)
Ever use	386	3,624	0.96 (0.82-1.12)
Long-term use	61	656	0.86 (0.62-1.20)
**Cumulative DDDs**			
Never use	486	5,096	1.00 (ref)
<500	262	2,352	1.00 (0.84-1.18)
≥500 - <1000	63	616	0.88 (0.66-1.18)
≥1000 - <1500	25	279	0.82 (0.53-1.26)
≥1500	36	377	0.86 (0.59-1.26)
OR per 500 DDDs	386	3,624	0.95 (0.87-1.03)
**Among women with inflammatory bowel diseases**			
**Use categories**			
Never use	446	4,459	1.00 (ref)
Ever use	231	2,311	0.92 (0.76-1.11)
Long-term use	22	282	0.70 (0.39-1.25)
**Cumulative DDDs**			
Never use	446	4,459	1.00 (ref)
<500	165	1,544	0.96 (0.78-1.17)
≥500 - <1000	44	485	0.81 (0.56-1.16)
≥1000 - <1500	9	152	0.55 (0.27-1.12)
≥1500	13	130	1.04 (0.56-1.96)
OR per 500 DDDs	231	2,311	0.92 (0.80-1.05)

Abbreviations: CI, confidence interval; DDD, defined daily dose; OR, Odds ratio.

[1] Adjusted for age and calendar time (by risk-set matching and the conditional analysis).

[2] Adjusted for age, calendar time (by risk-set matching and the conditional analysis), asthma, rheumatoid arthritis, polymyalgia rheumatica/ giant cell arthritis, psoriasis arthritis, ankylosing spondylitis, Crohn's disease, ulcerative colitis, renal diseases, multiple sclerosis, Charlson comorbidity index score, ever use of immunosuppressants, ever use of non-steroidal anti-inflammatory drugs, ever use of proton pump inhibitors, former and recent use of oral contraceptives and former and recent use of hormone replacement therapy. .

Appendix 1 – Danish Nationwide Health Registries

The **Danish Cancer Registry** has recorded incident cases of cancer on a nationwide basis since 1943 and provides accurate and almost complete records of cancer cases in Denmark. Cancer diagnoses are coded according to the *International Classification of Diseases*, *Tenth Revision* 10 (ICD-10) and the ICD for Oncology (ICD-O-1-3) for topography and morphology (26).

The **Danish Pathology Register** contains records of pathological specimens, which for some departments dates back as early as 1970 (30). Since 1990, all departments of pathology have used electronic registrations and from 1997, it became a legal obligation to report the pathologies to the Danish Pathology Register. The registry contains coded diagnoses based on the Danish Systematized Nomenclature of Medicine (SNOMED).

The **Danish National Prescription Registry** contains data on all prescription drugs filled by Danish residents since 1995. The data include the type of drug, date of filling, and quantity (27). The dosing information and the indication for prescribing are not available and no information is available on drugs used at hospital level. Drugs are categorized according to the Anatomic Therapeutic Chemical (ATC) index, a hierarchical classification system developed by the World Health Organization, and the quantity dispensed for each prescription is described by the number and strength of the pharmaceutical entities (*e.g.*, tablets), as well as defined daily doses (DDD).

The **Danish National Patient Registry** contains nationwide data on all non-psychiatric hospital admissions since 1977 and on ambulatory hospital contacts and psychiatric admissions since 1995 (28). Discharge/contact diagnoses have been coded according to ICD-8 from 1977 to 1993 and ICD-10 since 1994.

Statistics Denmark is a governmental institution that collects and processes information for a variety of statistical and scientific purposes, e.g. education and income. It hosts the **Population Education Registry,** which contains information on nearly all adult Danes and provides the highest completed level of education, defined as the longest duration of schooling (29).

The Danish **Civil Registration System** contains data on addresses, migration, and date of death (31,32). This system allowed us to extract population controls and to keep track of all subjects during the study period.

Appendix 2– Codes and definitions

**Breast cancer diagnosis, morphology and stage**		
**Breast cancer cases**	*ICD-10*	C50.0-C50.9
**Histological subtype**		
Ductal adenocarcinoma	*Morphology code*	85003, 85013, 85023, 85033, 85043, 85073, 85213, 85403, 85413
Lobular adenocarcinoma	*Morphology code*	85203, 85223
Other	*Morphology code*	81403, 82003, 82013, 82113, 82303, 82463, 82603, 83103, 83153, 84013, 84413, 84503, 84803, 84813, 84903, 85103, 85303, 85503, 85603, 85703, 85723, 85733, 85753, 80103, 80123, 80133, 80203, 80213, 80223, 80413, 80463, 80503, 80703, 80743, 80003, 80013, 87203, 88003, 88103, 88303, 88903, 89343, 89403, 89803, 89823, 90103, 90203, 91203, 91303, 95903, 95913, 96703, 96733, 96803, 96903, 97023
**Stage**		
Localized	*TNM*	(T=11-19 & N=30 & M=40) or (T=11-13 & [N=30 or N=39] & [M=40 or M=49]) or (T=14 & N=30 & M=49) or (T=14 & N=39 & M=40)
Non localized	*TNM*	(T=11-19 & N=31-33) or M=41
Others	*TNM*	(T=19 & N=39 & M=49) or none of the above
**ER status**		
ER+	Pathology code	F29521, FYY501
ER-	Pathology code	F29525, FYY505
**Exclusion criteria**		
Any cancer (except non-melanoma skin cancer)	*ICD-10*	C00-97 (except C44)
Mastectomy	*NCSP-code*	KHAC
**Systemic glucocorticoids**		H02AB
Betamethasone	*ATC code*	H02AB01
Dexamethasone	*ATC code*	H02AB02
Methylprednisolone	*ATC code*	H02AB04
Prednisolone	*ATC code*	H02AB06
Prednisone	*ATC code*	H02AB07
Triamcinolone	*ATC code*	H02AB08
Hydrocortisone	*ATC code*	H02AB09
**Other drugs**		
Immunossupressants	*ATC code*	L04
Nonsteroidal anti-inflammatory drygs	*ATC code*	M01A
Proton pump inhibitors	*ATC code*	A02BC02
Hormone replacement therapy excluding vaginally administered estrogens	*ATC code*	G03C, G03F, G03HB01 (excluding vaginally administered estrogens)
Raloxifen		G03XC01
Oral contraceptives	*ATC code*	G03A
**Prior diagnoses (diagnostic code or drug marker)**		
Alcohol-related diseases (proxy of heavy alcohol consumption)	*ICD-8*	291, 303, 425.5, 537.5, 571.0, 571.1, 571.2, 571.3, 577.10
	ICD-10	F10, E244, G312, G621, G721, I426, K292, K70, K852, K860, Q860, Z502 Z714, Z721
	ATC code	N07BB
Asthma	ICD-8	493
	ICD-10	J45, J46
Chronic obstructive pulmonary disease	ICD-8	491,492
	ICD-10	J41–J44
	ATC code	R03BB, R03AC
Rheumatoid arthritis	ICD-8	712.0 712.1, 712.2, 712.3 712.5
	ICD-10	M05-M06
Polymyalgia rheumatica/ Giant cell arthritis	ICD-8	446.30, 446.31, 446.39

	ICD-10	M315, M316, M35.3
Psoriasis arthritis	ICD-8	696.09
	ICD-10	M07.0-M07.3
Ankylosing spondylitis	ICD-8	712.4
	ICD-10	M45
Crohn's disease	ICD-8	563.01, 563.02, 563.09
	ICD-10	K50
Ulcerative colitis	ICD-8	563.1
	ICD-10	K51
Renal diseases	ICD-8	249.02, 250.02, 403, 404, 580-584, 590.09, 593.20, 753.10-753.19
	ICD-10	N00, N01, N03, N04, N05 N06, N07, N08, N11, N14, N15, N16, N17, N18, N19, N26, N27, N28, N29, I12. I13, I15.0, I15.1, E10.2, E11.2, E14.2, Q61.1-Q61.4
Multiple sclerosis	ICD-8	340
	ICD-10	G35
Adrenal insufficiency	ICD-8	253, 255.10, 255.11
	ICD-10	E23.0, E24.0, E27.1, E27.2, E27.4, E89.3
**Educational level**		
Basic	*Duration*	7-10 years
Medium	*Duration*	11–12 years
Higher	*Duration*	≥13 years
Unknown	*Duration*	-

NOTES:
ICD = International Classification of Disease
ATC = Anatomical Therapeutic Chemical
NCSP = Nordic Classification of Surgical procedures
ER= Estrogen Receptor
